# Epitope mapping of vaccine antigens Tc24 and TSA1 with antibodies from Trypanosoma cruzi infected patients

**DOI:** 10.21203/rs.3.rs-7216993/v1

**Published:** 2025-08-07

**Authors:** Eric Dumonteil, Claudia Herrera

**Affiliations:** Department of Tropical Medicine, Vector-Borne and Infectious Disease Research Center, School of Public Health and Tropical Medicine, Tulane University, New Orleans; School of Public Health and Tropical Medicine, Tulane University, New Orleans

## Abstract

Chagas disease is a zoonotic disease caused by *Trypanosoma cruzi* parasites. Tc24 and TSA1 parasite antigens are leading candidates for a therapeutic vaccine to treat infected patients to stop/delay the progression of chronic cardiomyopathy. As these antigens are nearing clinical trials, we aimed to assess their epitope recognition profile by antibodies from Chagas disease patients to better understand their immunogenicity in humans. Peptide microarrays covering Tc24-C4 and TSA1-C4 vaccine antigens were incubated with IgG from 27 *T. cruzi*-infected patients from Argentina, Honduras and Mexico. Most patients (20/27, 74%) had a highly similar recognition profile of both vaccine antigens, with the same immunodominant epitopes (three epitopes for Tc24-C4 and four for TSA1-C4). Remaining patients had limited reactivity against these antigens, targeting epitopes that varied among patients. All immunodominant epitopes were well conserved among *T. cruzi* strains and DTUs and most were accessible on the surface of the proteins. The immunodominant epitope recognition profile was observed independently of patient HLA profile, diagnostic test reactivity or *T. cruzi* parasite burden. Patients were infected with mixtures of TcI, TcII, TcIV, TcV and TcVI parasites. These results present an important baseline for assessing potential changes in epitope profiles following therapeutic vaccination in future clinical trials.

## Introduction

Chagas disease is a major parasitic disease in the Americas, affecting over 6 million patients with a large disease burden. Infection with *Trypanosoma cruzi*, the protozoan parasite responsible for the disease, can lead to chronic cardiomyopathy and/or digestive disease such as megaesophagus or megacolon many years after infection in 30–40% of cases, while the remainer of patients is asymptomatic, despite parasite persistence in all patients^1, 2^. Current treatments for infected patients are limited to benznidazole and nifurtimox, which are effective during the acute phase following infection, but their efficacy decreases during the chronic phase^3^. In addition, these drugs are associated with multiple side effects that add challenges to treatment completion^4–7^.

As part of recent efforts at developing a vaccine that may be used as an immunotherapeutic treatment, alone or combined with benznidazole, two parasite antigens have emerged as promising for their inclusion in a vaccine formulation^8^. Tc24 is a flagellar associated calcium binding protein^9, 10^ and TSA1 is part of the trans-sialidase family of multicopy genes encoding major surface proteins of the parasite^11^. Extensive preclinical studies have shown that recombinant Tc24-C4 and TSA1-C4, which underwent cysteine mutagenesis to improve solubility and expression^12–14^, can effectively treat infected mice, reducing parasite burden, cardiac inflammation and fibrosis^14–19^. Advanced studies in non-human primates^20–22^ and a first field trial in naturally infected dogs^23^ have further evidenced that vaccine treatment can preserve cardiac function, making this vaccine formulation attractive for the development of a human vaccine.

Remarkably, *T. cruzi* infected patients from Mexico were found to have antibodies against Tc24 and TSA1 antigens, and antigen-stimulated PBMCs from these patients were able to mount a recall response, indicating that both antigens may be immunogenic during natural infection in humans^24^. However, *T. cruzi* is genetically very diverse, being classified into seven major near-clade or discrete-typing units (DTUs) TcI to TcV and TcBat^25, 26^. While both antigens have been found to be highly conserved across DTUs^27, 28^, it is still unclear which specific epitopes of these antigens are being targeted by the host antibody response and how these may vary among human populations. As these antigens are nearing clinical trials, it is key to better understand their immunogenicity during natural *T. cruzi* infection.

Thus, our aim was to assess the epitope recognition profile of Tc24-C4 and TSA1-C4 using antibodies from Chagas disease patients from various countries to better understand the immunogenicity of these antigens in humans. We used peptide microarrays to map epitopes from these antigens using plasma samples, and the recognition profile was analyzed in the context of patient characteristics including diagnostic test results, HLA and the infecting *T. cruzi* parasite strains.

## Material and Methods

### Patient samples

The present study was approved by Tulane University institutional IRB (No. 2018–2237). De-identified archived samples were derived from a previous study on congenital *T. cruzi* transmission^29^ and consisted in maternal plasma samples collected at birth in Argentina (N = 5), Honduras (N = 5) and Mexico (N = 19), with a well characterized *T. cruzi* infection status based on Stat-Pak (Chembio Diagnostics) and T-detect (InBios) rapid tests and a recombinant ELISA (Wiener), as well as *T. cruzi* PCR (Table 1). Parasite burden was also measured by qPCR. Ten samples were reactive with 2–3 serological tests, of which 8 were PCR positive. Seventeen samples were serodiscordant (reactive with 0–1 serological tests), but PCR positive for *T. cruzi*. Two seronegative and PCR negative control samples from Mexico were also included. Positive and serodiscordant samples were equally distributed among the three countries (Table 1). IgG was purified from plasma samples using Thermo Scientific^™^ Melon^™^ Gel IgG Spin Purification Kit as instructed, and IgG concentration was measured on a Nanodrop2000 spectrophotometer.

### Peptide microarrays

The sequence from Tc24-C4 and TSA1-C4 (REFS) were used to generate overlapping 15-mers peptide, with an overlap of 13 amino acids covering these antigens. The Herpes envelope epitope SHRANETIYNTTLKY sequence was included as a positive control. Each unique peptide was synthesized in duplicate on a C-terminal — βAla — Asp spacer on glass slides by Schafer-N (Denmark) and Cy3 blank spots were also included as negative controls. Microarray slides were deprotected in TFA EDT H_2_0 for 3h at room temperature and blocked overnight in 0.1% BSA, 0.1% Tween-20 in PBS. After blocking, slides were incubated for 1h at room temperature with purified IgG from patients (100 μg/ml IgG in 0. 1% BSA, 0.1% Tween-20 in PBS), washed 3 × 20 min with 0.1% BSA, 0.1% Tween-20 in PBS and incubated for 1h at room temperature with Cy3- goat anti-Hu IgG (1 μg/ml in 0.1% B SA, 0.1% Tween-20 in PBS). Slides were washed 3 × 20 min with 0.1% BSA in PBS, dried, and scanned using a laser scanner with 1 μm resolution to measure fluorescent signal intensity^30^. IgG binding intensity to duplicate peptides was averaged to assess binding profile along antigen sequences.

### 3D modeling of epitopes

Epitopes were localized on the 3D structure of Tc24 (PDB ID 3CS1) and TSA1 AlphaFold prediction (Uniprot Q26971_TRYCR), to assess potential exposure to IgG in native antigens. Structures and epitopes were visualized in UCSF ChimeraX^31^.

### HLA typing and T. cruzi genotyping

HLA typing of patients was performed by CD Genomics for Class I alleles of HLA-A, -B and -C and Class II alleles of HLA-DPA1, -DPB1, -DQA1, -DQB1, -DRB1, and -DR345 loci, based on sequencing of the respective genes. Allele frequencies from the HLA genes were compared between groups of patients using C^2^ tests. Principal component analysis (PCA) was also performed to assess potential differences in HLA profile using the complete Class I and Class II gene set, or the Class II genes only, since these are thought to be more relevant for antibody responses^32^. Genotyping of *T. cruzi* was performed by deep sequencing of the mini-exon marker, that was PCR amplified as before^33, 34^.

### Epitope conservation among T. cruzi strains

Tc24 and TSA1 epitope conservation among *T. cruzi* strains and DTUs was assessed by BLASTp searches on a custom database from 32 *T. cruzi* genomes covering TcI (n = 12); TcII (n = 4); TcIII (n = 3); TcIV (n = 5); TcV (n = 3) and TcVI (n = 5)(Supplementary Table 1). Sequences from the epitopes identified in these genomes were aligned and visualized with WebLogo^35^. The proportion of genomes from each DTU in which epitopes were identified was also calculated.

## Results

**Figure 1:**
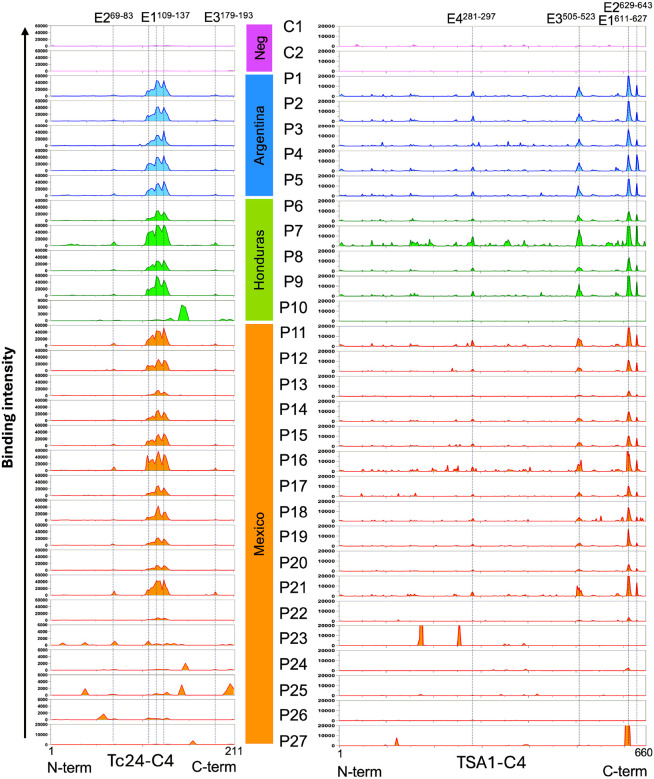
Epitope mapping of Tc24-C4 and TSA1-C4 vaccine antigens. Overlapping peptides covering the full length of the primary sequence for Tc24-C4 (left) and TSA1-C4 (right), horizontal axis, were evaluated in microarrays with IgGs from 27 individual Chagas disease patients from the indicated countries (P1-P27) and 2 negative controls (C1 and C2). The main epitopes (E1-E4) are indicated for each antigen.

Epitope mapping for Tc24-C4 and TSA1-C4 vaccine antigens was performed using overlapping peptide microarrays with IgG from 27 *T. cruzi* infected patients and two negative controls. As expected, IgG from negative controls showed no binding to these two antigens. On the other hand, IgG from most *T. cruzi* infected patients showed some recognition of both antigens, although some variability was detected among individuals (Fig. 1). For Tc24-C4, a strongly recognized region with several overlapping epitopes was observed in the middle of the protein for 20/27 patients (E1^109–137^), including patients from Argentina, Honduras and Mexico. Two weak but consistent epitopes were also observed on both sides of this immunodominant region, epitopes E2^69–83^ and E3^179–193^. The seven remaining patients presented weak or no IgG binding to the immunodominant epitopes, but some recognition of several other epitopes that mostly differed among individuals. One patient was from Honduras and six were from Mexico (Fig. 1). Two patients (P22 and P27) showed negligible reactivity to Tc24-C4.

For TSA1-C4, four dominant epitopes were identified for the same 20/27 patients strongly reacting to Tc24-C4, and TSA1-C4 epitopes were mostly localized in the C-terminus side of the protein (E1^611–627^; E2^629–643^; E3^505–523^; and E4^281–297^). Several minor epitopes also seemed to be consistently but weakly recognized by IgG from several of the patients. IgG from the other seven patients showed very weak recognition of TSA1-C4 with a few alternative epitopes in some individual samples, but three patients (P10, P25 and P26) also showed negligible recognition of this antigen (Fig. 1).

Together, these results suggested that there were two main types of antigen recognition profiles: most patients (20/27, 74%, consisting of P1-P9, P11-P21) had a highly similar recognition profile of both vaccine antigens, with the same immunodominant epitopes. On the other hand, IgGs from a minority of patients (7/27, 26%, consisting of P10, P22-P27) presented a low/absent recognition of these immunodominant epitopes, but most recognized alternative epitopes that varied among individuals.

**Figure 2:**
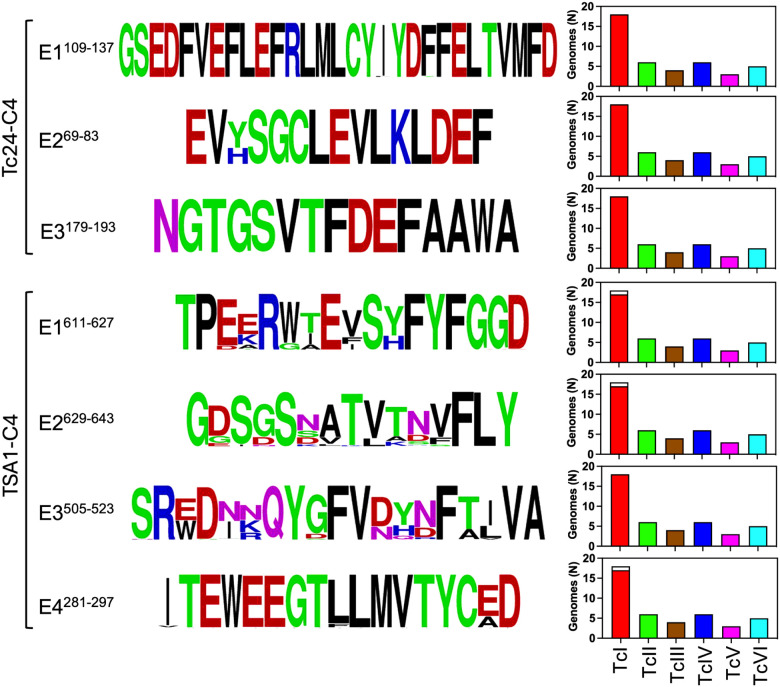
Epitope sequence conservation among *T. cruzi* strains. Sequence conservation of the indicated epitopes was visualized using Weblogo. Amino acids are color-coded according to their chemistry: Green= Polar; Purple= Neutral; Blue= Basic; Red= Acidic; Black= Hydrophobic. The bar graphs show the proportion of *T. cruzistrains* from DTUs TcI to TcVI in which the epitope was identified, with the full bars indicating strains in which it is present and the empty bars indicating strains in which it is absent.

Next, we assessed epitope sequence conservation among *T. cruzi* strains and DTUs. Both the immunodominant region and secondary Tc24-C4 epitopes were highly conserved with no/negligible amino acid substitutions, except in position 71 of epitope E2^69–83^ (Fig. 2). Tc24-C4 epitopes were also present in all parasite DTUs analyzed. For TSA1-C4, the four immunodominant epitopes were somewhat less conserved, except for epitope E4^281–297^ (Fig. 2). Nonetheless, the immunodominant sequences for these epitopes were the most frequent among parasite strains, and these were also detected in most strains and DTUs. The lack of some of the epitopes in one of the TcI strains likely reflected artefacts in genome sequencing/annotation rather than a true absence.

**Figure 3:**
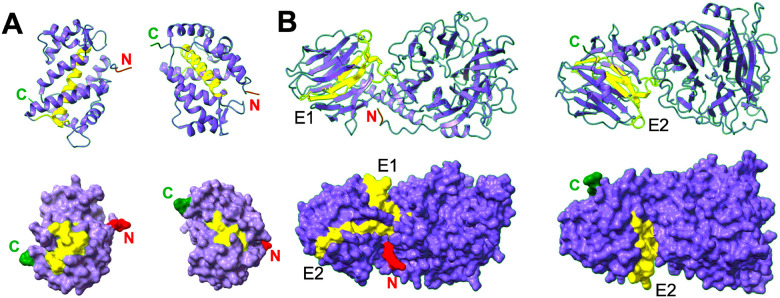
Localization of epitopes on antigen 3D structures. Epitopes (E1 to E4) from Tc24-C4 (**A**) and TSA1-C4 (**B**) are highlighted in colors in ribbons (top) and surface (bottom) renderings. The N terminus (red) and C-terminus (green) of the proteins are indicated.

Mapping of the epitopes on Tc24-C4 3D structure indicated that all three epitopes from this antigen were accessible on the surface of the proteins, except the N-terminus part of epitope E1^109–137^ domain, which corresponded to part of a a-helix inside the protein (Fig. 3A). While the epitope E1^109–137^ contained several overlapping epitopes, this N-terminus part appeared to be less strongly recognized by antibodies than the rest of the E1^109–137^ domain (Fig. 1). For TSA1-C4, the immunodominant epitopes E1^611–627^ and E2^629–643^ were well accessible on the surface of the proteins, while epitope E3^505–523^ was only partially exposed in a groove of the protein, and only the two loops flanking b-sheets from Epitope E4^281–297^ protruded on the surface of the protein (Fig. 3B). These data suggested that IgG may bind to most of these epitopes within native proteins, except for epitopes E3^505–523^ and E4^281–297^ from TSA1-C4, for which binding may be more constrained.

Because Chagas disease patients seemed to present two main types of antigen recognition profiles, we next assessed patient characteristics that may explain such differences. We first tested for potential differences in *T. cruzi* diagnostics (Table 1). There was no difference in reactivity for Stat-Pak (C^2^ = 0.005; d.f.=1; P = 0.94), T-detect (C^2^ = 0.59; d.f.=1; P = 0.44), ELISA (C^2^ = 0.38; d.f.=1; P = 0.54) tests, PCR assay (C^2^ = 0.58; d.f.=1; P = 0.45) reactivity or overall serodiscordance (C^2^ = 0.14; d.f.=1; P = 0.71) between the two groups of patients with different antigen recognition profiles (Supplementary Table 2). Similarly, optical density (OD) reading of the ELISA assays and blood parasite burden were not significantly different between the two patient groups (t = 0.2, P = 0.86 and t = 1.5, P = 0.24, respectively) (Supplementary Table 2). Thus, patient response to the various *T. cruzi* diagnostic assays had no influence on their vaccine epitope recognition profiles.

**Figure 4:**
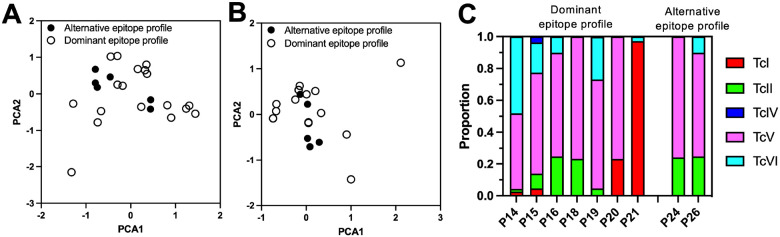
Patient HLA profile and infecting *T. cruzi* strains. PCA analysis of Class I and Class II (**A**) and only Class II HLA alles (**B**). Patients are grouped as with an immunodominant epitope profile (P1-P9, P11-P21, N=20), or as with an alternative epitope profile (P10, P22-P27, N=7). (**C**) DTU composition of *T. cruzi* strains infecting individual patients. The proportion of DTUs is shown, with DTU color-coded as indicated.

Since HLA is a major component of the immune response, we typed both Class I and Class II HLA from these patients and obtained reliable typing for 17 patients recognizing the immunodominant epitopes and 6 patients recognizing alternative epitopes. Comparison of HLA allele frequencies for individual genes indicated that there were no significant differences between the two groups of patients for any of the genes (Supplementary Table 3). Further, PCA analysis of individual HLA profiles also indicated a similar profile between the two groups (Fig. 4A), even when only Class II genes were analyzed (Fig. 4B). Thus, patient HLA profile did not explain the differences in their epitope recognition profiles of Tc24-C4 and TSA1-C4. Finally, we analyzed the contribution of the infecting *T. cruzi* strains. However, we were only able to genotype parasites from nine patients, seven presenting the immunodominant epitope profile, and two the alternative epitope profile (Fig. 4C). All patients were infected with mixtures of parasite DTUs including TcI, TcII, TcIV, TcV and TcVI in variable proportions, with no clear pattern suggesting potential differences in parasite composition among the two patient groups, possibly due to the very low sample size. Remarkably, even patients with the same immunodominant epitope profile were infected with a broad diversity of parasite DTUs in different proportions, although TcV predominated.

## Discussion

*T. cruzi* vaccine antigens Tc24-C4 and TSA1-C4 have been proposed as promising antigens for the development of an immunotherapeutic vaccine to stop or at least delay Chagas disease progression in infected humans^8, 36, 37^. In the context of forthcoming clinical trials, it is critical to better understand the immune response to these antigens during natural infections in diverse patient populations. We analyzed here the epitope recognition profile of these antigens by IgGs from patients from Argentina, Honduras and Mexico using overlapping peptide microarrays.

A first key observation was that most (74%) of patients presented the same epitope recognition profile for both Tc24-C4 and TSA1-C4, with 3–4 consistently reactive epitopes in each antigen. This recognition profile was observed independently of patient diagnostic tests results, parasite burden, HLA profile or infecting parasite DTUs. Indeed, patients presenting this antigen recognition profile were infected with diverse mixtures of TcI, TcII, TcIV, TcV and TcVI in variable proportions. Together, these results suggest a strong immunodominant epitope recognition profile resulting from natural infections with highly variable mixtures of *T. cruzi* parasite strains, across broad human populations. We recently proposed the term “cruziome” to refer to the multiple strains co-infecting a host, which we argued may be central to driving the host immune response and Chagas disease progression^38^, as observed in naturally infected macaques^39, 40^. It is thus striking that such diverse infections in different human populations produce such a consistent epitope recognition pattern of the two vaccine antigens.

One factor that may have contributed to this immunodominant epitope profile among most patients was the high level of sequence conservation of these epitopes across all parasite DTUs. Even epitopes from TSA1-C4, which were somewhat less conserved that those from Tc24, still presented limited variability of selected amino acid that may have allowed for sufficient cross-reactivity. Indeed, antibody binding intensity to the epitopes resulted variable among patients, suggesting individual differences in affinity or antibody levels targeting these epitopes. As TSA1 is part of the large family of trans-sialidase multicopy genes, many of which may be simultaneously expressed during infection^41, 42^, epitopes from different proteins but with sufficient sequence similarity may be targeted by cross-reactive antibodies.

Analysis of the 3D structure of the antigens indicated that epitope E1^109–137^ from Tc24 covers most of the second EF hand calcium binding domain (EF-2) and E3^179–193^ coincides with most of EF-4^43^, which can explain their high sequence conservation. Furthermore, all three Tc24-C4 epitopes regions have been found to be under purifying selection, and epitopes E1^109–137^ and E2^69–83^ overlap with several HLA class I epitopes^27^.

For TSA1-C4 antigen, epitope E2^629–643^ includes the trans-sialidase VTVxNVxLYNR signature motif^44, 45^. Epitope E1^611–627^ has some similarity with a trans-sialidase immunodominant epitope (cluster 32 − 3) identified with phage-display library screening with patient sera^46^, and a murine immunodominant and partially protective CD8^+^ epitope overlaps with E3^505–523 47, 48^. On the other hand, all IgG epitopes identified here differed from HLA class I epitopes from TSA1^28^, as well as from previously identified^49, 50^ or predicted^51^ epitopes from other members of the trans-sialidase family. Except for epitopes E3^505–523^ and E4^281–297^ from TSA1-C4, for which binding may be more constrained, all other epitopes appeared to be readily accessible on the surface of the 3D structure on the antigens, suggesting that IgGs may be able to bind to native proteins. This is also encouraging as vaccination with the recombinant antigens may also target these epitopes^21, 22^, although this remains to be confirmed in future studies.

Despite the consistent epitope recognition profile in most patients, a minority of patients (26%) showed a different profile, with limited/no recognition of the immunodominant epitopes, and some recognition of alternative epitopes that varied among individuals, with IgGs from a few individuals showing no recognition of either antigen. This agrees with previous studies indicating that most but not all patients have antibodies against Tc24 and TSA1^9, 24^. Similarly in dogs, about only about 80–93% of *T. cruzi* infected dogs have antibodies against these antigens^52, 53^. However, it is unclear why these few patients presented such a different antibody profile against the vaccine antigens, as this did not appear to be associated with their overall immune response to infection, as assessed by the different serological diagnostic tests, nor with the parasite burden, nor their HLA profile. Our data are too limited to completely rule out parasite strains, but all patients for which parasite genotyping was successful harbored very diverse “cruziomes” as mentioned above.

In conclusion, we identified major epitopes from TC24-C4 and TSA1-C4 vaccine antigens recognized by IgGs from *T. cruzi* infected patients following natural infections with mixtures of parasite strains from TcI, TcII, TcIV, TcV and TcVI DTUs. Most patients presented an immunodominant epitope recognition profile of both antigens, independently of their HLA profile, diagnostic test reactivity or *T. cruzi* parasite burden. These epitopes are conserved among the six DTUs frequently infecting humans. These results present an important baseline for assessing changes in epitope profiles following therapeutic vaccination in future clinical trials.

## Supplementary Material

Supplementary Files

This is a list of supplementary files associated with this preprint. Click to download.

- SupplementaryTable1.docx
- SupplementaryTable2.docx
- SupplementaryTable3.docx

## Figures and Tables

**Table 1:** Patient characteristics

ID	Country	Stat-Pak	T -detect	ELISA	ELISA OD^$^	PCR	Parasite burden*
P1	Argentina	−	−	−	0.093	+	1.477
P2	Argentina	+	−	−	0	+	4.984
P3	Argentina	+	+	+	2.574	+	14.443
P4	Argentina	+	−	−	0.002	+	2.544
P5	Argentina	+	+	+	2.841	+	4.506
P6	Honduras	+	+	+	2.329	+	5.528
P7	Honduras	+	+	+	1.965	+	8.591
P8	Honduras	−	−	−	0.042	+	5.994
P9	Honduras	+	−	−	0.045	+	3.455
P10	Honduras	+	−	−	0.043	+	6.057
P11	Mexico	+	+	−	0.099	−	0
P12	Mexico	+	+	+	2.644	+	0.973
P13	Mexico	+	+	+	1.556	+	1.469
P14	Mexico	+	−	−	0.026	+	5.233
P15	Mexico	−	+	−	0.03	+	5.288
P16	Mexico	+	−	−	0.029	+	2.789
P17	Mexico	−	+	−	0.037	+	4.154
P18	Mexico	−	+	−	0.039	+	1.157
P19	Mexico	+	−	−	0.041	+	2.207
P20	Mexico	−	+	−	0.037	+	4.101
P21	Mexico	+	−	−	0.034	+	2.837
P22	Mexico	+	−	−	0.043	+	1.34
P23	Mexico	+	+	+	0.777	−	0
P24	Mexico	−	+	−	0.032	+	3.066
P25	Mexico	+	+	+	2.174	+	3.502
P26	Mexico	−	+	−	0.047	+	2.333
P27	Mexico	+	+	+	2.561	+	1.47
N1	Mexico	−	−	−	0.035	−	ND
N2	Mexico	−	−	−	0.035	−	ND

$ optical density.

* parasite equivalent/ml of blood. ND: not done.

## Data Availability

All data generated or analysed during this study are included in this published article and its supplementary information files.
